# Indirect treatment comparison of cabazitaxel for patients with metastatic castrate-resistant prostate cancer who have been previously treated with a docetaxel-containing regimen

**DOI:** 10.1371/journal.pone.0195790

**Published:** 2018-04-11

**Authors:** Jon P. Fryzek, Heidi Reichert, Nicholas Summers, Lindsay Townes, Robert Deuson, Dominik D. Alexander, Jackie Vanderpuye-Orgle

**Affiliations:** 1 EpidStat Institute, Ann Arbor, Michigan, United States of America; 2 Precision Health Economics, Los Angeles, California, United States of America; 3 Medenomics, LLC, Moorpark, California, United States of America; Texas Technical University Health Sciences Center, UNITED STATES

## Abstract

**Background:**

The objective of this study was to conduct an indirect treatment comparison between cabazitaxel, abiraterone and enzalutamide to determine the clinical efficacy and safety of cabazitaxel relative to comparators in the treatment of patients with metastatic castrate-resistant prostate cancer who progress on docetaxel-based therapies.

**Methods:**

A systematic literature review was conducted to inform the network meta-analysis of cabazitaxel, abiraterone and enzalutamide. Due to a lack of head-to-head trials, studies with a comparator arm of best supportive care were included in the analysis. Overall survival, progression-free survival, and adverse events were compared within both Bayesian and Frequentist frameworks. The ratios for survival outcomes were estimated using hazard ratios (HR), and the ratios for adverse events between groups were estimated using odds ratios (ORs); uncertainty was reported as 95% confidence (Frequentist) and credible (Baysesian) Intervals.

**Results:**

Three of thirteen trials identified for abstraction were relevant for analyses. Median overall survival was not statistically significantly different for abiraterone (HR = 1.04; 95% CI = 0.83–1.28) or enzalutamide (HR = 0.88; 95% CI = 0.69–1.11) when compared to cabazitaxel in the Bayesian analysis. Anaemia (OR = 3.71; 95% CI = 1.01–10.44), diarrhoea (OR = 16.60; 95% CI = 1.41–75.31) and haematuria (OR = 3.88; 95% CI = 1.03–10.09) were more likely to occur in the cabazitaxel group than the abiraterone group, while pyrexia risk was higher in cabazitaxel compared to enzalutamide (OR = 36.23; 95% CI = 1.14–206.40). Frequentist analyses produced similar results.

**Conclusions:**

The scarcity of clinical studies and lack of a common comparator limited analyses. The adverse event results must be interpreted with caution as many were based on small numbers. The results from this analysis indicate comparable survival outcomes and adverse event profiles. As these pivotal studies may not reflect the contemporary treatment landscape and patient profiles, additional research, including head-to-head clinical trials and real world observational studies, should be conducted to further elucidate the beneficial effects of these therapies.

## Introduction

Despite evolution in the treatment paradigm over the past decade, prostate cancer remains a significant public health burden. According to the American Cancer Society’s estimates, in 2016, approximately 180,890 new cases of prostate cancer were diagnosed in the United States, and about 26,120 men died of prostate cancer. It is estimated that 1 in 7 men will be diagnosed with prostate cancer during his lifetime [1]. In the United Kingdom, approximately 47,300 incident cases of prostate cancer occurred in 2013, with an estimated 11,287 deaths from this malignancy in 2014 [2].

Health Technology Assessment agencies often need to make new drug coverage decisions despite incomplete data on the comparative safety and efficacy of available treatment options. More often than not, newly approved drugs lack head-to-head comparison data from clinical trials for all available treatment options, limiting comparators to one or two legacy treatments. Due to concerns around adequate assessments of comparative effectiveness, global reimbursement agencies such as the United Kingdom’s National Institute for Health and Care Excellence (NICE), and more recently, major private payers in the United States, require alternative approaches for assessing comparative risk and efficacy be performed [3].

The challenge of inadequate comparative efficacy and safety data is prevalent in clinical studies of treatments for metastatic castrate-resistant prostate cancer (mCRPC). In particular, as new therapies were approved for this difficult to treat population, no direct head-to-head comparisons for novel therapies versus cabazitaxel have become available. Therefore, this study was undertaken to assess the comparative effectiveness and safety of cabazitaxel in patients with mCRPC who have been treated with docetaxel-based regimens relative to therapies endorsed by the latest guidelines in the United Kingdom for this indication (e.g., abiraterone and enzalutamide).

## Materials and methods

This indirect treatment comparison follows the PRISMA extension statement for reporting of systematic reviews incorporating network meta-analyses [4].

### Eligibility criteria

Phase 2 or Phase 3 randomized controlled trials with any blinding status of adult (age 18 or older) mCRPC patients previously treated with docetaxel-based regimens were included.

### Systematic literature review

**Fig 1:**
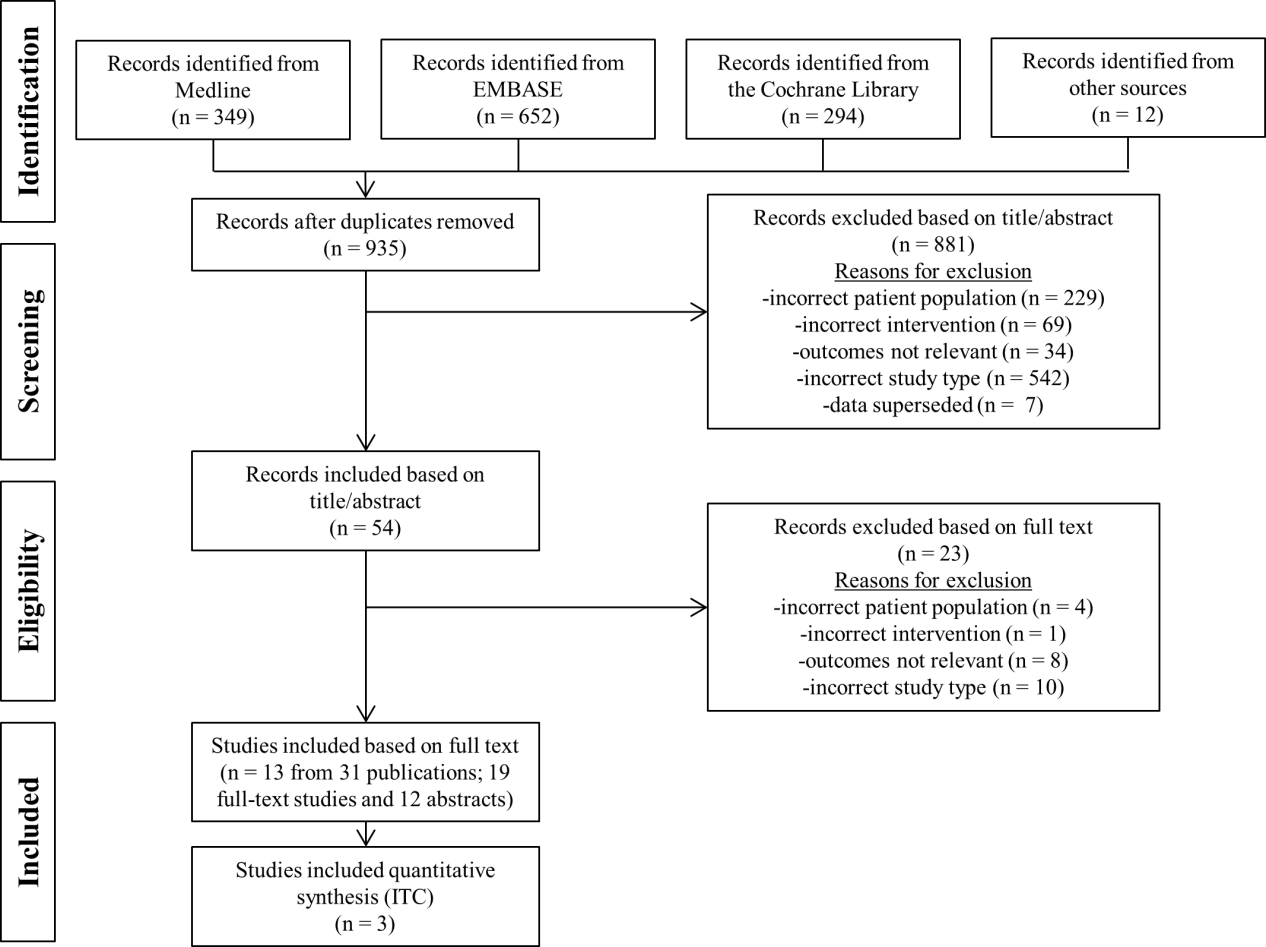
PRISMA trial flow diagram.

Results of the systematic literature review are available in a separate publication [5]. A formal protocol is not available for the systematic review. Details on the eligibility criteria, information sources or databases, key words and search strings utilized for the databases, study selection criteria, data collection process, and qualitative information and quantitative data extracted are presented in the prior publication. Database searches of MEDLINE, Embase, and Cochrane CENTRAL were conducted from January 1, 2010 to February 26, 2015 (see S1 Appendix for complete search strategies). Manual searches were conducted for abstracts from multiple congresses held between January 1, 2011 and March 8, 2015 –American Society of Clinical Oncology (ASCO); ASCO-Genitourinary; European Society for Medical Oncology; American Urological Association; American Association for Cancer Research; European Association of Urology; and Société Internationale d’Urologie. Two reviewers screened articles for inclusion using a two-step process whereby article titles and abstracts were screened, followed by full-text review. Any disputes about the abstracted data were resolved through discussion between reviewers or consultation with a third reviewer. To be eligible for inclusion, studies were required to have a population of patients with mCRPC who had received a docetaxel-based regimen for any previous line of therapy, and who were treated by one or more interventions of interest (cabazitaxel, abiraterone, enzalutamide, mitoxantrone, ipilimumab, radium-223, sipuleucel-T, and estramustine). A pre-specified list of relevant efficacy and safety outcomes, such as overall survival (OS), progression-free survival (PFS) data and adverse events (AE), were required to be reported. The PRISMA flow diagram is illustrated in Fig 1.

Data from relevant articles were extracted in parallel by two independent reviewers, and studies were critically examined using both a qualitative appraisal and a study grade. Both sets of extracted data were compared and combined into a final data extraction table, which was subsequently verified for the accuracy of all content by an independent third reviewer. In instances where multiple publications were identified for the same trial, the novel data reported in each publication were initially extracted separately and then grouped together to create the most complete dataset for each study population while avoiding double counting of the patients. Both qualitative study characteristics and quantitative data were extracted from each study. This enabled us to perform a systematic and critical evaluation of study relevancy and design homogeneity as described in the sections below. Variables abstracted included the following: study acronym and authors, country, randomization, blinding, control, line of therapy, age, sample size (%), length of follow-up, ECOG/WHO performance status, race, prior therapy, OS rate, PFS rate, and grade 3 or 4 AEs.

### Geometry of the network

In order to conduct the analyses, the first step was to synthesize the relevant studies by developing a map (network geometry) that details the specific direct comparisons, indirect comparisons, and comparisons of analytical interest across the trials. Before constructing a network, it is critical to review each study’s characteristics, methodology, and analytical comparisons and techniques in an effort to assemble a harmonized group of studies that could be evaluated collectively in a meta-analytic framework. Only studies that do not present significant heterogeneity relative to the other studies were included in the final analysis. The network depicts each drug in the analysis as a node, where the links between different nodes represent trials comparing the connected agents.

### Risk of bias within individual studies

Studies were assessed by performing semi-quantitative evaluations, qualitative appraisals, and study quality grading. Various components of the included studies were assessed for the possible introduction of bias using guidance adapted from the University of York’s Centre for Reviews and Dissemination [6]. Examples of the evaluated study criteria include appropriate patient randomization technique, double-blind study design and description of blinding methods, and adequate explanation of withdrawals and drop-outs. An overall qualitative study appraisal was also conducted using the Jadad score [7]. Additional information can be found in the systematic literature review manuscript [5].

Both a qualitative assessment and an analysis of statistical heterogeneity were used to determine the risk of bias during the study selection process. As indicated in the previous section, it is of critical importance to systematically examine the qualitative study appraisals for potential “design heterogeneity” or “conceptual heterogeneity” before it can be included in the quantitative assessment. Design heterogeneity is evaluated by qualitatively examining the study specific characteristics, such as the nature of the study population (e.g., age and gender), duration of follow-up, drop-out rate, and the sample size among other important characteristics. To evaluate statistical heterogeneity, statistical testing is performed to evaluate the magnitude and degree of between-study variability. By conducting an in-depth review of these study parameters, we were able to determine if there were substantial differences between the studies that would prevent the combining of quantitative data.

### Analytic methods

The outcomes of interest in these analyses included median OS, median PFS, and risk of various AEs. The measure of association for OS and PFS was the hazard ratio (HR) with 95% Confidence Intervals (95% CI) for Frequentist analyses and 95% Credible Intervals (also referred to as 95% CI henceforth) for Bayesian analyses. The ratios of AEs between groups were estimated using odds ratios (ORs) with 95% CIs. The monitoring scheme for PFS differed among the included studies; therefore, these analyses should be considered exploratory. The conduct of specific analyses was limited to the available data and to the scientific justification for including similar studies together in a quantitative fashion. A network meta-analysis comparing the effect of treatment with cabazitaxel to its comparators was performed within a Frequentist and a Bayesian framework, offering two different methodological approaches to the analyses [8]. These two analytical methodologies were utilized for the purpose of evaluating the consistency of findings. To address possible heterogeneity between studies, fixed and random effects models were used.

NICE recommends that vague or flat priors, such as N (0, 1002) be used for Bayesian analyses if there are a minimal number of clinical trials with large numbers of subjects [9]. Given that only one study for each drug of interest was identified in the systematic literature review, only a single model was built. The Deviance Information Criterion (DIC) was used to assess “goodness of fit” of the model. Statistical heterogeneity was measured with the I^2^ statistic.

Frequentist analyses were conducted in Stata [10], and Bayesian analyses were performed using WinBugs based on code supplied by Woods et al., 2010 [11].

### Assessment of inconsistency

Inconsistency can be assessed using statistical methodology when the network geography contains closed loops. However, we were not able to evaluate inconsistency because there is no available literature that accommodates closed loops for these treatments.

### Risk of bias across studies

Factors that may differ across studies and lead to bias include the characteristics of the patients (e.g., age distribution), the way in which the outcomes are measured (e.g., HRs for OS vs. proportions for disease free progression), the length of follow-up (e.g., comparable evaluations based on variable lengths of patient follow-up), and the study timeframe.

## Results

### Study selection

The comprehensive literature search produced 935 studies that underwent initial screening. After careful review for eligibility, 13 studies were fully abstracted from 31 publications. A flow diagram of the studies included and excluded at each stage is provided in Fig 1.

Only three of the clinical trials (AFFIRM, COU-AA-301, and TROPIC) had similar comparator groups. Thus, the analyses focused on these trials. Results for the TROPIC study came from Joulain et al (2010) [12], results for AFFIRM were published by Scher et al (2012) [13], and results for COU-AA-301 were reported by Fizazi (2012) [14].

### Summary of network geometry

**Fig 2:**
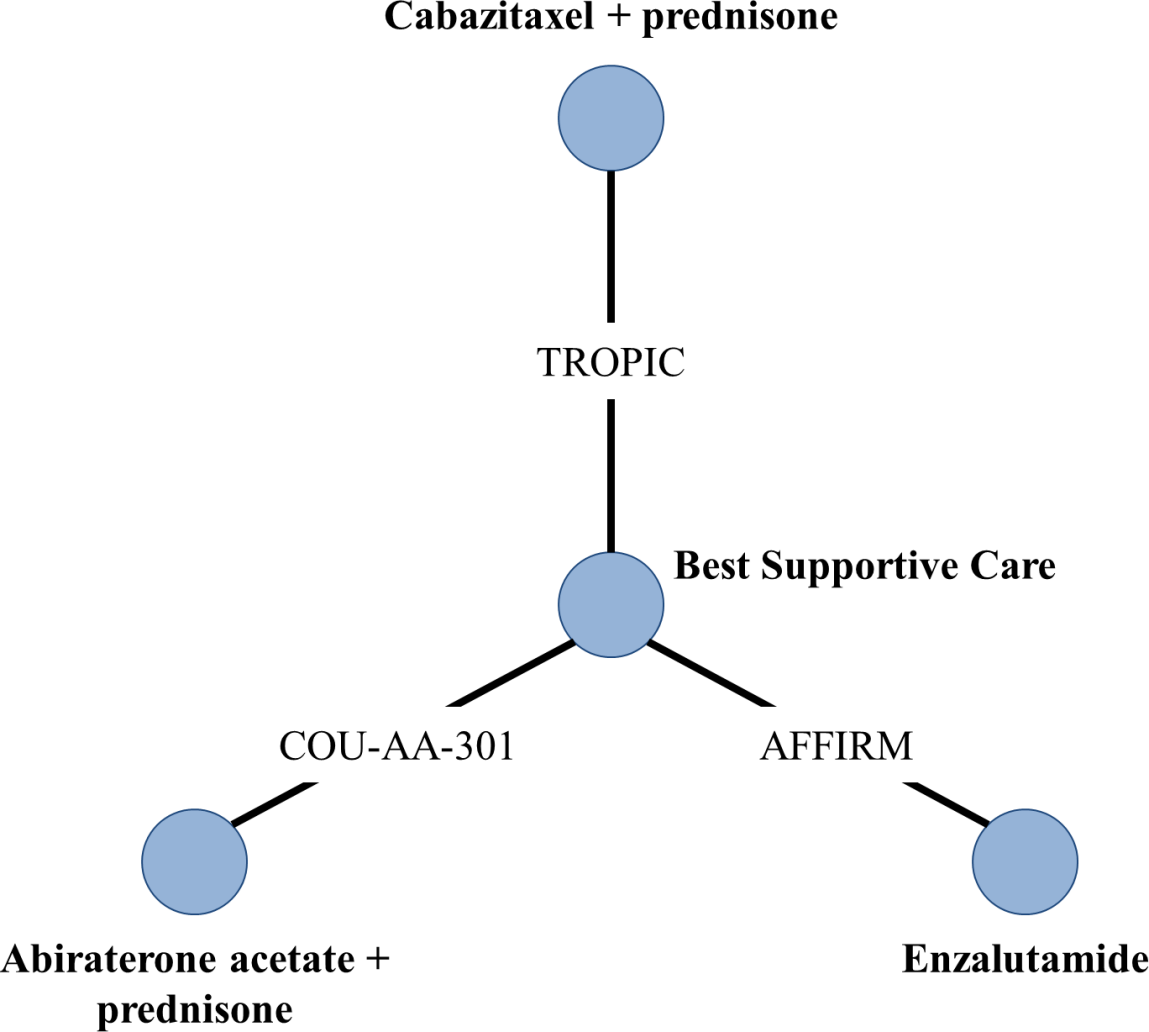
Network geometry of published randomized studies. Direct comparisons = solid line/link between the nodes; best supportive care varied by study: TROPIC = mitoxantrone + prednisone, COU-AA-301 = prednisone, and AFFIRM = placebo.

The treatment network was based on three clinical trials. For the drugs of interest, no direct comparisons could be made. Therefore, all comparisons were made indirectly through their relationship with a common comparator (Fig 2).

For these analyses, it was assumed that comparator patients in all three trials received best supportive care. Furthermore, we assumed that the placebo used in the comparator group in the AFFIRM trial had no relationship to any of our outcomes (OS, PFS, AEs). The COU-AA-301 trial used prednisone to treat their comparator patients, while TROPIC used mitoxantrone and prednisone. A recent study showed that survival patterns were similar for prednisone and mitoxantrone/prednisone treated groups; although, AEs may be more abundant in the mitoxantrone/prednisone group among mCRPC patients [15]. Therefore, the indirect treatment comparison of cabazitaxel, abiraterone, and enzalutamide is anchored on best supportive care.

### Study characteristics

Table 1 compares the characteristics for each study included in the indirect treatment comparisons. The studies are similar in terms of treatment line (pre-treated with docetaxel-based regimen), study years (range: 2007–2010), total patients (range: 755–1199), age (median range: 68–69) and ECOG score (0–1 range: 90%-93%).

**Table 1:** Baseline characteristics of the studies included in the indirect treatment comparison for cabazitaxel, abiraterone acetate and enzalutamide.

Author (Year)	Acronym	TX Line	Study Type	Treatment /	Study Years	Region	Total Patients	Age	Race	ECOG
				Comparator						
Joulain (2010)	TROPIC	3	RCT	Cabazitaxel + prednisone /	2007–2008	26 countries	755	≥75 yrs: 18% median: 68	White: 83.5% Asian: 7.5% Black: 5% Other: 3.5%	0–1: 93%
				Mitoxantrone + prednisone						
Fizazi (2012)	COU-AA-301	3	RCT	Abiraterone acetate + prednisone /	2008–2009	13 countries	1195	≥75 yrs: 28% median: 69	NR	0–1: 90% 2: 10%
				Placebo + prednisone						
Scher (2012)	AFFIRM	3	RCT	Enzalutamide /	2009–2010	15 countries	1199	≥75 yrs: 25.3% median: 69	NR	0–1: 91.5% 2: 8.5%
				Placebo						

RCT = Randomized controlled trial; TX = Treatment

### Results of individual studies

Individual study results for median OS are reported in Table 2. Similar median lengths of study follow-up were found for the TROPIC and COU-AA-301 studies (i.e., 623.96 days and 614.82 days, respectively). The median length of follow-up for the AFFIRM study was slightly less at 428.29 days. The active arm of all studies had better median survival compared to best supportive care.

**Table 2:** Hazard ratios (HR) and 95% Confidence Interval (CI) for overall survival (OS) for studies included in the indirect treatment comparison for cabazitaxel, abiraterone acetate and enzalutamide.

Author (Year)	Acronym	Treatment / Comparator	Length of follow-up (median)	Median OS HR (95% CI)	HR p-value
Joulain (2010)	TROPIC	Cabazitaxel + prednisone /	623.96 days	0.72 (0.61–0.84)	<0.0001
		Mitoxantrone + prednisone			
Fizazi (2012)	COU-AA-301	Abiraterone acetate + prednisone /	614.82 days	0.74 (0.64–0.86)	<0.0001
		Placebo + prednisone			
Scher (2012)	AFFIRM	Enzalutamide /	438.29 days	0.63 (0.53–0.75)	<0.001
		Placebo			

Median PFS is reported in Table 3 along with the definitions used to measure PFS in each study. These differences may contribute to the considerably shorter median length of follow-up for the TROPIC study compared to COU-AA-301 and AFFIRM since TROPIC uses a broader definition of the PFS endpoint. As with OS, PFS was statistically significantly longer in the active arm compared to best supportive care in all three trials.

**Table 3:** Hazard ratios (HR) and 95% Confidence Interval (CI) for progression-free survival (PFS) for studies included in the indirect treatment comparison for cabazitaxel, abiraterone acetate and enzalutamide.

Study	Treatment	Length of follow-up (median)	Median PFS HR (95% CI; p-value)	Description of PFS Endpoint
TROPIC	Cabazitaxel + prednisone	85 days	0.75 (0.65–0.87; <0.0001)	The earliest progression in tumor, PSA or pain or death
	Mitoxantrone + prednisone	43 days		
COU-AA-301	Abiraterone acetate + prednisone	170.45 days	0.66 (0.58–0.76; <0.0001)	Soft-tissue disease progression by modified Response Evaluation Criteria In Solid Tumors (RECIST) criteria
	Placebo + prednisone	109.57 days		
AFFIRM	Enzalutamide	252.63 days	0.40 (0.35–0.47; <0.001)	Progression of soft-tissue disease according to RECIST, version 1.1, progression of osseous disease according to bone scans showing two or more new lesions per PCWG2, and death from any cause.

The proportion of individuals with AEs graded 3 or higher for each study is reported in Table 4. Adverse events in the TROPIC trials were ascertained from the Sanofi internal report [12] for all AEs except cardiac disorders and abnormalities in liver function tests (available on clinicaltrials.gov for trial NCT00417079). All AEs identified in the COU-AA-301 trials were published in Fizazi (2012) [14], while AEs from the AFFIRM trials were mainly identified from clinicaltrials.gov for trial NCT00974311, except for diarrhoea, fatigue, cardiac disorder and abnormalities in liver function tests, which were available in Scher (2012) [13]. The most common AEs among cabazitaxel users were neutropenia (21.3%), febrile neutropenia (7.5%), and diarrhoea (6.2%). The most common AEs among abiraterone were fatigue (9.5%), followed by anaemia (8%) and back pain (7.5%). For enzalutamide, fatigue (6%), anaemia (2.63%), haematuria (1.5%), and bone pain (1.5%) were most common.

**Table 4:** Percent grade 3 and above adverse events (AEs) for cabazitaxel, abiraterone acetate and enzalutamide and their comparators.

**CABAZITAXEL (TROPIC)**				
**Adverse Event**	**Author (year)**	**Follow-up**	**Cabazitaxel % with AE**	**Comparator % with AE**
Abdominal pain	TROPIC (2010)	max: 3 yrs / med: 20.5 mos	1.9%	0%
Abnormalities in liver function tests	NCT00417079 (2011)	max: 2 yrs	0.3%	0%
Anaemia	TROPIC (2010)	max: 3 yrs / med: 20.5 mos	3.5%	1.3%
Arthralgia	TROPIC (2010)	max: 3 yrs / med: 20.5 mos	1.1%	1.1%
Asthenia	TROPIC (2010)	max: 3 yrs / med: 20.5 mos	4.6%	2.4%
Back pain	TROPIC (2010)	max: 3 yrs / med: 20.5 mos	3.8%	3%
Bone pain	TROPIC (2010)	max: 3 yrs / med: 20.5 mos	0.8%	2.4%
Cardiac disorders	NCT00417079 (2011)	max: 2 yrs	1.89%	0.81%
Constipation	TROPIC (2010)	max: 3 yrs / med: 20.5 mos	1.1%	0.5%
Diarrhoea	TROPIC (2010)	max: 3 yrs / med: 20.5 mos	6.2%	0.3%
Dyspnoea	TROPIC (2010)	max: 3 yrs / med: 20.5 mos	1.3%	0.8%
Fatigue	TROPIC (2010)	max: 3 yrs / med: 20.5 mos	4.9%	3%
Febrile neutropenia	TROPIC (2010)	max: 3 yrs / med: 20.5 mos	7.5%	1.3%
Haematuria	TROPIC (2010)	max: 3 yrs / med: 20.5 mos	1.9%	0.5%
Nausea	TROPIC (2010)	max: 3 yrs / med: 20.5 mos	1.9%	0.3%
Neutropenia	TROPIC (2010)	max: 3 yrs / med: 20.5 mos	21.3%	7%
Pain	TROPIC (2010)	max: 3 yrs / med: 20.5 mos	1.1%	1.9%
Pain in extremity	TROPIC (2010)	max: 3 yrs / med: 20.5 mos	1.6%	1.1%
Pyrexia	TROPIC (2010)	max: 3 yrs / med: 20.5 mos	1.1%	0.3%
Thrombocytopenia	TROPIC (2010)	max: 3 yrs / med: 20.5 mos	2.4%	0.3%
Urinary-tract infection	TROPIC (2010)	max: 3 yrs / med: 20.5 mos	1.1%	0.8%
Vomiting	TROPIC (2010)	max: 3 yrs / med: 20.5 mos	1.9%	0%
**ABIRATERONE (COU-AA-301)**				
**Adverse Event**	**Author (year)**	**Follow-up**	**Abiraterone % with AE**	**Comparator % with AE**
Abdominal pain	Fizazi (2012)	max: 2 yrs / med: 20.2 mos	2%	2%
Abnormalities in liver function tests	Fizazi (2012)	max: 2 yrs / med: 20.2 mos	4.5%	3.5%
Anaemia	Fizazi (2012)	max: 2 yrs / med: 20.2 mos	8%	9%
Arthralgia	Fizazi (2012)	max: 2 yrs / med: 20.2 mos	5%	4%
Asthenia	Fizazi (2012)	max: 2 yrs / med: 20.2 mos	3%	2.5%
Back pain	Fizazi (2012)	max: 2 yrs / med: 20.2 mos	7.5%	10.5%
Bone pain	Fizazi (2012)	max: 2 yrs / med: 20.2 mos	6.5%	8%
Cardiac disorders	Fizazi (2012)	max: 2 yrs / med: 20.2 mos	5%	2.5%
Constipation	Fizazi (2012)	max: 2 yrs / med: 20.2 mos	1%	1%
Diarrhoea	Fizazi (2012)	max: 2 yrs / med: 20.2 mos	1.5%	1%
Dyspnoea	Fizazi (2012)	max: 2 yrs / med: 20.2 mos	2.5%	2.5%
Fatigue	Fizazi (2012)	max: 2 yrs / med: 20.2 mos	9.5%	10.5%
Febrile neutropenia	Fizazi (2012)	max: 2 yrs / med: 20.2 mos	0.5%	0%
Haematuria	Fizazi (2012)	max: 2 yrs / med: 20.2 mos	2%	2%
Nausea	Fizazi (2012)	max: 2 yrs / med: 20.2 mos	2.5%	3%
Neutropenia	Fizazi (2012)	max: 2 yrs / med: 20.2 mos	0.5%	0.5%
Pain	Fizazi (2012)	max: 2 yrs / med: 20.2 mos	0.5%	2.5%
Pain in extremity	Fizazi (2012)	max: 2 yrs / med: 20.2 mos	3.5%	5%
Pyrexia	Fizazi (2012)	max: 2 yrs /med: 20.2 mos	0.5%	1%
Thrombocytopenia	Fizazi (2012)	max: 2 yrs / med: 20.2 mos	1.5%	1%
Urinary-tract infection	Fizazi (2012)	max: 2 yrs / med: 20.2 mos	2%	0.5%
Vomiting	Fizazi (2012)	max: 2 yrs / med: 20.2 mos	3.5%	3%
**ENZALUTAMIDE (AFFIRM)**				
**Adverse Event**	**Author (year)**	**Follow-up**	**Enzalutamide % with AE**	**Comparator % with AE**
Abdominal pain	NCT00974311 (2014)	max: 3 yrs	0.13%	0.5%
Abnormalities in liver function tests	Scher (2012)	max: 2 yrs / med: 14.4 mos	0.5%	0.5%
Anaemia	NCT00974311 (2014)	max: 3 yrs	2.63%	3.01%
Arthralgia	NCT00974311 (2014)	max: 3 yrs	0.38%	0.25%
Asthenia	NCT00974311 (2014)	max: 3 yrs	0.38%	0.75%
Back pain	NCT00974311 (2014)	max: 3 yrs	1.38%	1.75%
Bone pain	NCT00974311 (2014)	max: 3 yrs	1.5%	1%
Cardiac disorders	Scher (2012)	max: 2 yrs / med: 14.4 mos	1%	2%
Constipation	NCT00974311 (2014)	max: 3 yrs	0.63%	0.75%
Diarrhoea	Scher (2012)	max: 2 yrs / med: 14.4 mos	1.0%	0.5%
Dyspnoea	NCT00974311 (2014)	max: 3 yrs	0.13%	0.25%
Fatigue	Scher (2012)	max: 2 yrs / med: 14.4 mos	6.0%	7%
Febrile neutropenia	Not Available			
Haematuria	NCT00974311 (2014)	max: 3 yrs	1.50%	1.25%
Nausea	NCT00974311 (2014)	max: 3 yrs	0.63%	0.75%
Neutropenia	Not Available			
Pain	NCT00974311 (2014)	max: 3 yrs	0.63%	0.25%
Pain in extremity	NCT00974311 (2014)	max: 3 yrs	0.38%	0.5%
Pyrexia	NCT00974311 (2014)	max: 3 yrs	0.25%	1.25%
Thrombocytopenia	NCT00974311 (2014)	max: 3 yrs	0.13%	0%
Urinary-tract infection	NCT00974311 (2014)	max: 3 yrs	0.88%	1.25%
Vomiting	NCT00974311 (2014)	max: 3 yrs	0.25%	2.01%

### Indirect treatment comparison results

#### Overall survival

We used the TROPIC, COU-AA-301, and AFFIRM trials to compare cabazitaxel with abiraterone and enzalutamide indirectly through a common comparator using a Bayesian fixed effects model. No statistically significant difference in median OS for patients treated with abiraterone was observed when compared with patients treated with cabazitaxel (HR = 1.04; 95% CI = 0.83–1.28). While patients on enzalutamide had a better median OS compared to cabazitaxel, this difference was not statistically significant (HR = 0.88; 95% CI = 0.69–1.11) (Table 5). For this analysis, statistical heterogeneity was not significant (I^2^ 3.1% (0%-89.9%)) and the DIC was low (DIC = 5.93).

**Table 5:** Median overall survival and progression-free survival hazard ratios (HRs) and 95% Credible Intervals for cabazitaxel, abiraterone and enzalutamide using Bayesian fixed effects modeling.

	Hazard Ratio	95% Credible Interval
**Overall Survival**		
Cabazitaxel vs comparator	0.72	0.61–0.85
Abiraterone vs comparator	0.74	0.64–0.86
Enzalutamide vs comparator	0.63	0.53–0.75
Abiraterone vs Cabazitaxel	1.04	0.83–1.28
Enzalutamide vs Cabazitaxel	0.88	0.69–1.11
Enzalutamide vs Abiraterone	0.86	0.68–1.07
**Progression-Free Survival**		
Cabazitaxel vs comparator	0.75	0.65–0.87
Abiraterone vs comparator	0.66	0.58–0.76
Abiraterone vs Cabazitaxel	0.88	0.72–1.07

#### Progression-free survival

We compared the median PFS of patients on abiraterone to patients on cabazitaxel indirectly through a common comparator using a Bayesian fixed effects model. Data from the AFFIRM trial was excluded because the confidence interval for PFS did not overlap the confidence interval of the two other trials, indicating heterogeneity. PFS was modestly lower for abiraterone than cabazitaxel, but the difference was not statistically significant (HR = 0.88; 95% CI = 0.72–1.07) (Table 5).

#### Adverse events

The results for the AEs must be interpreted with caution as many of these outcomes were based on very few events resulting in unstable risk estimates. Of some of the more frequently reported AEs in the 3 included trials (e.g., fatigue, anaemia, back pain, diarrhoea), only anaemia (OR = 3.71; 95% CI = 1.01–10.44) and diarrhoea (OR = 16.60; 95% CI = 1.41–75.31) were statistically significantly more likely to occur in the cabazitaxel group compared to abiraterone (Table 6). In addition, the indirect treatment comparison showed haematuria (OR = 3.88; 95% CI = 1.03–10.09) and pyrexia (OR = 36.23; 95% CI = 1.14–206.40) were higher among those receiving cabazitaxel compared to those receiving abiraterone and enzalutamide, respectively. None of the other AEs were statistically significantly different for the three groups.

**Table 6:** Odds ratios and 95% Credible Intervals for grade 3 and above adverse events.

Adverse Event	Cabazitaxel vs Abiraterone*		Cabazitaxel vs Enzalutamide*	
	Odds Ratios	95% Credible Intervals	Odds Ratios	95% Credible Intervals
Anaemia	3.71	1.01–10.44	3.99	0.89–12.24
Arthralgia	1.07	0.16–3.79	2.68	0.10–13.86
Asthenia	2.01	0.55–5.27	6.99	0.63–29.56
Back pain	2.04	0.76–4.50	1.95	0.44–5.54
Bone pain	0.48	0.07–1.45	0.27	0.56–1.03
Cardiac disorders	1.74	0.26–6.58	8.04	0.95–32.86
Constipation	4.28	0.24–21.05	5.63	0.24–29.43
Diarrhoea	16.60	1.41–75.31	11.44	0.55–56.73
Dyspnoea	2.59	0.32–10.13	72.84	0.09–178.30
Fatigue	2.01	0.79–4.33	2.13	0.79–4.76
Haematuria	3.88	1.03–10.09	0.81	0.16–2.43
Nausea	3.05	0.83–7.79	0.17	0.04–0.47
Neutropenia	5.14	0.42–19.98	NA	NA
Pain	4.49	0.50–17.75	0.69	0.04–3.09
Pain in extremity	3.00	0.54–10.08	2.11	0.03–11.74
Pyrexia	9.92	0.46–52.19	36.23	1.14–206.40
Thrombocytopenia	6.61	0.48–31.99	NA	NA
Urinary-tract infection	0.53	0.03–2.48	3.32	0.26–14.83

Note: all omitted AEs had a least one cell with 0 observations

*Referent (or comparison) group

### Risk of bias across studies

Based on our assessment of design heterogeneity, we did not identify potential sources of qualitative variability or bias that would preclude the combination of quantitative data across studies. Because only a few studies were deemed relevant for the quantitative meta-analysis, we were able to efficiently compare and contrast the study design and characteristics between studies. Specifically, for OS, important study characteristics, such as age, ECOG score, and length of follow-up were relatively similar between studies. The TROPIC study had an expanded definition for a progression-free endpoint, which may have shortened PFS compared to AFFIRM or COU-AA-301. A broad overview of comparisons of the three trials is in Table 7.

**Table 7:** Comparison of age, ECOG score, and lengths of follow-up for overall survival (OS) and progression-free survival (PFS) for clinical trials used in the indirect treatment comparison.

Study	Total Patients	Age	ECOG	Length of OS follow-up (median)	Length of PFS follow-up (median)	Description of PFS
TROPIC	755	≥ 75 yrs: 18%; median: 68	0–1: 93%	623.96 days	Treatment: 85 days	The earliest progression in tumor, PSA or pain or death
					Comparator: 43 days	
COU-AA-301	1195	Treatment: ≥75 yrs: 25%; median: 69	Treatment: 0–1: 91%; 2: 9%	614.82 days	Treatment: 170.45 days	Soft-tissue disease progression by modified Response Evaluation Criteria In Solid Tumors (RECIST) criteria
		Comparator: ≥75 yrs: 26%; median: 69	Comparator: 0–1: 92%; 2: 8%		Comparator: 109.57 days	
AFFIRM	1199	Treatment: ≥75 yrs: 24.9%	0–1: 91.5%; 2: 8.5%	438.29 days	Treatment: 252.63 days	Progression of soft-tissue disease according to RECIST, version 1.1, progression of osseous disease according to bone scans showing two or more new lesions per PCWG2, and death from any cause.
		Comparator: ≥75 yrs: 26.1%			Comparator: 88.27 day	

### Additional analyses

We repeated analyses using a Frequentist model, a Bayesian fixed effects model, and a Bayesian random effects model to understand the robustness of the results for overall survival. It should be noted that the posterior distributions of the Bayesian random effects model are skewed, and thus in the presence of skewed distributions, the median was used for inference. The three approaches produced similar findings (Table 8).

**Table 8:** Hazard ratios and 95% Credible Intervals for indirect treatment comparisons between cabazitaxel, abiraterone and enzalutamide using Bayesian fixed and random effects and Frequentist fixed effects modeling.

Approach	Hazard Ratio	95% Credible Interval
**Bayesian Fixed Effects**		
Cabazitaxel vs comparator	0.72	0.61–0.85
Abiraterone vs comparator	0.74	0.64–0.86
Enzalutamide vs comparator	0.63	0.53–0.75
Abiraterone vs Cabazitaxel	1.04	0.83–1.28
Enzalutamide vs Cabazitaxel	0.88	0.69–1.11
Enzalutamide vs Abiraterone	0.86	0.68–1.07
**Bayesian Random Effects**		
Cabazitaxel vs comparator	0.71	0.21–2.43
Abiraterone vs comparator	0.74	0.22–2.50
Enzalutamide vs comparator	0.62	0.19–2.16
Abiraterone vs Cabazitaxel	1.03	0.29–3.60
Enzalutamide vs Cabazitaxel	0.87	0.25–3.14
Enzalutamide vs Abiraterone	0.85	0.24–3.05
**Frequentist Fixed Effects**		
Cabazitaxel vs comparator	0.72	0.61–0.84
Abiraterone vs comparator	0.74	0.64–0.86
Enzalutamide vs comparator	0.63	0.53–0.75
Abiraterone vs Cabazitaxel	1.03	0.83–1.28
Enzalutamide vs Cabazitaxel	0.88	0.69–1.11
Enzalutamide vs Abiraterone	0.85	0.68–1.07

## Discussion

### Summary of evidence

Median OS was similar for cabazitaxel, abiraterone and enzalutamide. While median PFS was modestly lower for abiraterone than cabazitaxel, the results of the exploratory analysis were not statistically significant. Anaemia, diarrhoea and haematuria were statistically significantly more likely to occur in the cabazitaxel group compared to abiraterone, while only pyrexia was higher among those receiving cabazitaxel compared to those receiving enzalutamide. None of the other AEs were statistically significantly different for the three groups.

### Comparison to past studies

Four studies have conducted indirect treatment comparisons between abiraterone and enzalutamide for overall survival [16–19], but these did not consider cabazitaxel in the analysis. Two of the past studies [18, 19] supported our findings for the indirect estimate of OS between abiraterone and enzalutamide (HR = 0.86; 95% CI 0.68–1.07). In modest contrast, an HR of 0.97 (95% CI = 0.75–1.25) for OS was reported in a study by Brodszky [16]. It appears that this study included an interim report [20] and a final report [14] from the COU-AA-301 trial for abiraterone in their indirect treatment comparisons which may have skewed their results. One study [17] compared mean survival for the two groups and found that the mean survival for enzalutamide (38.7 months; 95% CI = 36.4–40.7) was greater than the mean survival for abiraterone (34.6 months; 95% CI = 31.8–37.8). However, the mean survival time is not informative for understanding the efficacy of a drug on survival as all of the deaths in the study population would have to be observed before a valid mean survival time could be calculated. Indirect estimates of median radiographic PFS for abiraterone and enzalutamide were similar (both calculated an indirect HR = 0.61; 95% CI = 0.50–0.74) in two studies [18, 19] while a third study compared means of radiographic PFS [17]. We did not include enzalutamide in our indirect treatment comparison analysis of PFS as the AFFIRM trial introduced heterogeneity into the model. While three of the studies [16, 18, 19] also evaluated AEs, to our knowledge, our study was the first to formally compare these outcomes for abiraterone, enzalutamide and cabazitaxel.

### Limitations

The main limitation of these analyses is the lack of a specified common comparator. While evidence exists showing that survival among prostate cancer patients taking mitoxantrone/prednisone and prednisone alone is similar [15], there is no evidence to support that PFS is similar in these groups. The PFS analyses should be considered exploratory as the studies did not have a consistent definition for PFS. The results for the analysis of AEs must be interpreted with caution as well since patients treated with mitoxantrone/prednisone may experience different rates of safety outcomes than those treated with prednisone alone [15]. Additionally, many of the specific AEs were based on very small numbers resulting in unstable risk estimates, and follow-up time varied between studies. The scarcity of clinical studies also limited the types and numbers of analyses that could be performed, and brings with it the inability to conduct or rely on the findings of the underlying tests of publication bias and study heterogeneity.

In order to conduct an indirect treatment comparison, treatments are compared through their comparators. Given this, we were only able to identify three registrational trials conducted pre-marketing to include in our study. In essence, the comparative assessment methodology was limited to randomized controlled trial-defined data reporting under circumstances that do not reflect current therapeutic sequencing and utilization patterns that are based on additional scientific evidence generated from post-marketing trials and observational studies. The addition of new studies may change findings reported here.

## Conclusions

In this analysis of pivotal clinical trial data, cabazitaxel, abiraterone and enzalutamide had similar survival outcomes and AE profiles. To the best of our knowledge, this is the first indirect treatment comparison including these 3 agents evaluating both efficacy and safety outcomes. Data from future trials should be incorporated into this study framework to garner additional information about the relative performance of these drugs.

## Supporting information

S1 Table PRISMA checklist. (DOCX)

S1 Appendix Complete search strategy. (DOCX)
