# Supplementary material for: Indirect treatment comparison of cabazitaxel for patients with metastatic castrate-resistant prostate cancer who have been previously treated with a docetaxel-containing regimen
Source: PLoS One. 2018 Apr 11;13(4):e0195790. doi: 10.1371/journal.pone.0195790 (PMC5895064; doi:10.1371/journal.pone.0195790)
Supplement: S1 Appendix — (DOCX) [file pone.0195790.s002.docx]

# S1 Appendix. Complete search strategy

Databases searches were conducted on February 26, 2015.

Embase/MEDLINE RCT searches (via Embase.com)

| **No.** | **Query** |
| --- | --- |
| #1 | 'controlled study'/exp OR 'controlled study' |
| #2 | 'clinical trial'/exp OR 'clinical trial' OR 'clinical trials' |
| #3 | 'controlled clinical trial'/exp OR 'controlled clinical trial' |
| #4 | 'randomized controlled trial'/exp OR 'randomized controlled trial' OR 'randomised controlled trial' OR 'randomized controlled trials' OR 'randomised controlled trials' |
| #5 | 'randomisation'/exp OR 'randomisation' |
| #6 | 'randomization'/exp OR 'randomization' |
| #7 | 'random allocation'/exp OR 'random allocation' |
| #8 | 'randomly allocated' |
| #9 | 'allocated randomly' |
| #10 | rct |
| #11 | allocated NEAR/2 random |
| #12 | 'placebo'/exp OR 'placebo' OR placebo* |
| #13 | 'single-blind procedure'/exp OR 'single-blind procedure' |
| #14 | 'double-blind procedure'/exp OR 'double-blind procedure' |
| #15 | 'crossover procedure'/exp OR 'crossover procedure' |
| #16 | 'triple-blind procedure'/exp OR 'triple-blind procedure' |
| #17 | (single OR double OR triple OR treble) NEAR/1 (blind* OR mask*) |
| #18 | 'prospective study'/exp OR 'prospective study' |
| #19 | #1 OR #2 OR #3 OR #4 OR #5 OR #6 OR #7 OR #8 OR #9 OR #10 OR #11 OR #12 OR #13 OR #14 OR #15 OR #16 OR #17 OR #18 |
| #20 | 'case study'/exp |
| #21 | 'case report' |
| #22 | 'abstract report'/exp |
| #23 | 'letter'/exp |
| #24 | #20 OR #21 OR #22 OR #23 |
| #25 | #19 NOT #24 |
| #26 | cancer* OR carcinoma* OR malignan* OR tumo?r* OR neoplas* OR adeno* |
| #27 | hormone NEAR/3 (resistant OR refractory) |
| #28 | castrat* NEAR/3 resistant |
| #29 | #26 AND #27 |
| #30 | #26 AND #28 |
| #31 | 'prostate cancer'/exp OR 'prostate cancer' |
| #32 | 'prostate tumour'/exp OR 'prostate tumor' |
| #33 | prostat* NEAR/3 (cancer* OR carcinoma* OR malignan* OR tumo?r* OR neoplasm* OR adeno* OR intraepithelial) |
| #34 | hrpc OR crpc |
| #35 | #29 OR #30 OR #31 OR #32 OR #33 OR #34 |
| #36 | #25 AND #35 |
| #37 | #25 AND #35 AND [2010-2015]/py |
| #38 | (docetaxel OR taxotere OR taxane) NEAR/4 (prior OR previous* OR past OR earlier OR former OR pretreat* OR initial*) |
| #39 | (docetaxel OR taxotere OR taxane) NEAR/4 (after OR post OR following OR subsequent OR successive) |
| #40 | 'pre-treated' OR 'pretreated' OR 'prior therapy' |
| #41 | 're-challenge' |
| #42 | 'docetaxel-refractory' |
| #43 | 'taxane-refractory' |
| #44 | (treat* OR therapy OR chemotherapy) NEAR/1 (prior OR previous*) |
| #45 | '2nd line' OR 'second line' |
| #46 | '3rd line' OR 'third line' |
| #47 | '4th line' OR 'fourth line' |
| #48 | '5th line' OR 'fifth line' |
| #49 | '6th line' OR 'sixth line' |
| #50 | '7th line' OR 'seventh line' |
| #51 | '8th line' OR 'eighth line' |
| #52 | #38 OR #39 OR #40 OR #41 OR #42 OR #43 OR #44 OR #45 OR #46 OR #47 OR #48 OR #49 OR #50 OR #51 |
| #53 | #37 AND #52 |
| #54 | cabazitaxel OR jevtana OR 'xrp 6258' |
| #55 | dexamethasone |
| #56 | ipilimumab OR yervoy OR 'mdx 010' OR 'mdx 101' |
| #57 | estramustine OR emcyt OR estracit |
| #58 | mitoxantrone OR dhad OR novantrone |
| #59 | 'sipuleucel-t' OR provenge OR apc8015 |
| #60 | abiraterone OR zytiga |
| #61 | enzalutamide OR xtandi OR mdv3100 |
| #62 | 'radium-223' OR 'ra-223' OR alpharadin OR xofigo |
| #63 | #54 OR #55 OR #56 OR #57 OR #58 OR #59 OR #60 OR #61 OR #62 |
| #64 | #53 AND #63 |

MEDLINE In-Process RCT searches (via PubMed)

| **No.** | **Query** |
| --- | --- |
| #1 | randomised controlled trial |
| #2 | clinical trial |
| #3 | phase I OR phase 1 OR phase II or phase 2 OR phase III OR phase 3 OR phase IV or phase 4 |
| #4 | randomisation OR randomised |
| #5 | random* allocat* |
| #6 | allocated randomly |
| #7 | allocated AND random* |
| #8 | RCT |
| #9 | placebo* |
| #10 | ((singl* or doubl* or treb* or tripl*) AND (blind* or mask*)) |
| #11 | #1 OR #2 OR #3 OR #4 OR #5 OR #6 OR #7 OR #8 OR #9 OR #10 |
| #12 | case report |
| #13 | historical article |
| #14 | letter |
| #15 | #12 OR #13 OR #14 |
| #16 | #11 NOT #15 |
| #17 | cancer* OR carcinoma* OR malignan* OR tumor* OR tumour* OR neoplas* OR adeno* |
| #18 | hormone AND (resistant OR refractory) |
| #19 | castrat* AND resistant |
| #20 | #17 AND #18 |
| #21 | #17 AND #19 |
| #22 | prostate cancer |
| #23 | prostate tumor OR prostate tumour |
| #24 | prostat* AND (cancer* OR carcinoma* OR malignan* OR tumor* OR tumour* OR neoplasm* OR adeno* OR intraepithelial) |
| #25 | HRPC OR CRPC |
| #26 | #20 OR #21 OR #22 OR #23 OR #24 OR #25 |
| #27 | #16 AND #26 |
| #28 | #16 AND #26 Filters: Publication date from 2010/01/01 to 2015/12/31 |
| #29 | (docetaxel OR taxotere OR taxane) AND (prior OR previous* OR past OR earlier OR former OR pretreat* OR initial*) |
| #30 | (docetaxel OR taxotere OR taxane) AND (after OR post OR following OR subsequent OR successive) |
| #31 | pre-treated OR pretreated OR prior therapy |
| #32 | re-challenge |
| #33 | docetaxel-refractory |
| #34 | taxane-refractory |
| #35 | (treat* OR therapy OR chemotherapy) AND (prior OR previous*) |
| #36 | 2nd line OR second line |
| #37 | 3rd line OR third line |
| #38 | 4th line OR fourth line |
| #39 | 5th line OR fifth line |
| #40 | 6th line OR sixth line |
| #41 | 7th line OR seventh line |
| #42 | 8th line OR eighth line |
| #43 | #29 OR #30 OR #31 OR #32 OR #33 OR #34 OR #35 OR #36 OR #37 OR #38 OR #39 OR #40 OR #41 OR #42 |
| #44 | #28 AND #43 |
| #45 | cabazitaxel OR jevtana OR xrp 6258 |
| #46 | dexamethasone |
| #47 | ipilimumab OR yervoy OR mdx 010 OR mdx 101 |
| #48 | estramustine OR emcyt OR estracit |
| #49 | mitoxantrone OR dhad OR novantrone |
| #50 | sipuleucel-t OR provenge OR apc8015 |
| #51 | abiraterone OR zytiga |
| #52 | enzalutamide OR xtandi OR mdv3100 |
| #53 | radium-223 OR ra-223 OR alpharadin OR xofigo |
| #54 | #45 OR #46 OR #47 OR #48 OR #49 OR #50 OR #51 OR #52 OR #53 |
| #55 | #44 AND #54 |

Cochrane CENTRAL searches

| **No.** | **Query** |
| --- | --- |
| #1 | cancer* or carcinoma* or malignan* or tumor* or tumour* or neoplas* or adeno* in Trials |
| #2 | hormone and (resistant or refractory) in Trials |
| #3 | castrat* and resistant in Trials |
| #4 | #1 and #2 |
| #5 | #1 and #3 |
| #6 | MeSH descriptor: [Prostatic Neoplasms] this term only |
| #7 | prostate cancer in Trials |
| #8 | prostate tumor or prostate tumour in Trials |
| #9 | prostat* and (cancer* or carcinoma* or malignan* or tumor* or tumour* or neoplasm* or adeno* or intraepithelial) in Trials |
| #10 | HRPC or CRPC |
| #11 | #1 or #2 or #3 or #4 or #5 or #6 or #7 or #8 or #9 or #10 |
| #12 | (docetaxel or taxotere or taxane) and (prior or previous* or past or earlier or former or pretreat* or initial*) in Trials |
| #13 | (docetaxel or taxotere or taxane) and (after or post or following or subsequent or successive) in Trials |
| #14 | pre-treated or pretreated or prior therapy in Trials |
| #15 | re-challenge in Trials |
| #16 | docetaxel-refractory in Trials |
| #17 | taxane-refractory in Trials |
| #18 | (treat* or therapy or chemotherapy) and (prior or previous*) in Trials |
| #19 | 2nd line or second line in Trials |
| #20 | 3rd line or third line in Trials |
| #21 | 4th line or fourth line in Trials |
| #22 | 5th line or fifth line in Trials |
| #23 | 7th line or seventh line in Trials |
| #24 | 8th line or eighth line in Trials |
| #25 | #12 or #13 or #14 or #15 or #16 or #17 or #18 or #19 or #20 or #21 or #22 or #23 or #24 |
| #26 | #11 and #25 |
| #27 | #11 and #25 Publication Year from 2010 to 2015 |
| #28 | cabazitaxel or jevtana or xrp 6258 in Trials |
| #29 | Dexamethasone in Trials |
| #30 | ipilimumab or yervoy or mdx 010 or mdx 101 in Trials |
| #31 | estramustine or emcyt or estracit in Trials |
| #32 | mitoxantrone or dhad or novantrone in Trials |
| #33 | sipuleucel-t or provenge or apc8015 in Trials |
| #34 | abiraterone or zytiga in Trials |
| #35 | enzalutamide or xtandi or mdv3100 in Trials |
| #36 | radium-223 or ra-223 or alpharadin or xofigo in Trials |
| #37 | #28 or #29 or #30 or #31 or #32 or #33 or #34 or #35 or #36 |
| #38 | #27 and #37 |
